# Hematopoietic Cell Transplantation in Patients With Primary Immune Regulatory Disorders (PIRD): A Primary Immune Deficiency Treatment Consortium (PIDTC) Survey

**DOI:** 10.3389/fimmu.2020.00239

**Published:** 2020-02-21

**Authors:** Alice Y. Chan, Jennifer W. Leiding, Xuerong Liu, Brent R. Logan, Lauri M. Burroughs, Eric J. Allenspach, Suzanne Skoda-Smith, Gulbu Uzel, Luigi D. Notarangelo, Mary Slatter, Andrew R. Gennery, Angela R. Smith, Sung-Yun Pai, Michael B. Jordan, Rebecca A. Marsh, Morton J. Cowan, Christopher C. Dvorak, John A. Craddock, Susan E. Prockop, Shanmuganathan Chandrakasan, Neena Kapoor, Rebecca H. Buckley, Suhag Parikh, Deepak Chellapandian, Benjamin R. Oshrine, Jeffrey J. Bednarski, Megan A. Cooper, Shalini Shenoy, Blachy J. Davila Saldana, Lisa R. Forbes, Caridad Martinez, Elie Haddad, David C. Shyr, Karin Chen, Kathleen E. Sullivan, Jennifer Heimall, Nicola Wright, Monica Bhatia, Geoffrey D. E. Cuvelier, Frederick D. Goldman, Isabelle Meyts, Holly K. Miller, Markus G. Seidel, Mark T. Vander Lugt, Rosa Bacchetta, Katja G. Weinacht, Jeffrey R. Andolina, Emi Caywood, Hey Chong, Maria Teresa de la Morena, Victor M. Aquino, Evan Shereck, Jolan E. Walter, Morna J. Dorsey, Christine M. Seroogy, Linda M. Griffith, Donald B. Kohn, Jennifer M. Puck, Michael A. Pulsipher, Troy R. Torgerson

**Affiliations:** ^1^Division of Pediatric Allergy, Immunology, BMT, Benioff Children's Hospital, University of California, San Francisco, San Francisco, CA, United States; ^2^Department of Pediatrics, Johns Hopkins All Children's Hospital, University of South Florida, St. Petersburg, FL, United States; ^3^Division of Biostatistics, Medical College of Wisconsin, Milwaukee, WI, United States; ^4^Department of Pediatrics, Fred Hutchinson Cancer Research Center, Seattle Children's Hospital, University of Washington School of Medicine, Seattle, WA, United States; ^5^Department of Pediatrics, Seattle Children's Hospital, University of Washington School of Medicine, Seattle, WA, United States; ^6^Laboratory of Clinical Infectious Diseases, National Institute of Allergy and Infectious Diseases, National Institutes of Health, Bethesda, MD, United States; ^7^Primary Immunodeficiency Group, Paediatric Immunology and Haematopoietic Stem Cell Transplantation, Translational and Clinical Research Institute, Great North Childrens' Hospital, Newcastle University, Newcastle upon Tyne, United Kingdom; ^8^Pediatric Blood and Marrow Transplant, University of Minnesota, Minneapolis, MN, United States; ^9^Division of Pediatric Hematology-Oncology, Boston Children's Hospital, Boston, MA, United States; ^10^Department of Pediatric Oncology, Dana-Farber Cancer Institute, Boston, MA, United States; ^11^Department of Pediatrics, Harvard Medical School, Boston, MA, United States; ^12^Division of Bone Marrow Transplantation and Immune Deficiency, Department of Pediatrics, Cincinnati Children's Hospital Medical Center, University of Cincinnati, Cincinnati, OH, United States; ^13^Texas Children's Cancer Center, Center for Cell and Gene Therapy, Baylor College of Medicine, Houston, TX, United States; ^14^Stem Cell Transplant and Cellular Therapy Service, Department of Pediatrics, Memorial Sloan Kettering Cancer Center, New York, NY, United States; ^15^Division of Bone Marrow Transplant, Aflac Cancer and Blood Disorders Center, Children's Healthcare of Atlanta, Emory University School of Medicine, Atlanta, GA, United States; ^16^Section of Transplantation and Cellular Therapy, Cancer and Blood Disease Institute, Keck School of Medicine, Children's Hospital Los Angeles, University of Southern California, Los Angeles, CA, United States; ^17^Departments of Pediatrics and Immunology, Duke University School of Medicine, Durham, NC, United States; ^18^Cancer and Blood Disorders Institute, Blood and Marrow Transplant Program, Johns Hopkins All Children's Hospital, St. Petersburg, FL, United States; ^19^Department of Pediatrics, Washington University School of Medicine, St. Louis, MO, United States; ^20^Division of Blood and Marrow Transplantation, Children's National Health System, George Washington University School of Medicine and Health Sciences, Washington, DC, United States; ^21^Department of Pediatrics, Immunology, Allergy, and Retrovirology Baylor College of Medicine, Texas Children's Hospital William T. Shearer Center for Human Immunobiology, Houston, TX, United States; ^22^Center for Cell and Gene Therapy, Baylor College of Medicine, Texas Children's Hospital Cancer Center, Houston, TX, United States; ^23^Department of Pediatrics, University of Montreal, Montreal, QC, Canada; ^24^Department of Pediatrics, University of Utah School of Medicine, Salt Lake City, UT, United States; ^25^Children's Hospital of Philadelphia, Perelman School of Medicine at University of Pennsylvania, Philadelphia, PA, United States; ^26^Department of Pediatrics, Alberta Children's Hospital, University of Calgary, Calgary, AB, Canada; ^27^Pediatric Stem Cell Transplantation, Columbia University College of Physicians and Surgeons, New York, NY, United States; ^28^Manitoba Blood and Marrow Transplant Program, CancerCare Manitoba, University of Manitoba, Winnipeg, MB, Canada; ^29^Department of Pediatrics, University of Alabama at Birmingham, Birmingham, AL, United States; ^30^Laboratory of Inborn Errors of Immunity, Department of Immunology, Microbiology and Transplantation, KU Leuven, Leuven, Belgium; ^31^Department of Pediatrics, University Hospitals Leuven, Leuven, Belgium; ^32^Phoenix Children's Hospital, Phoenix, AZ, United States; ^33^Research Unit for Pediatric Hematology and Immunology, Department of Pediatrics and Adolescent Medicine, Medical University Graz, Graz, Austria; ^34^Department of Pediatrics, University of Michigan, Ann Arbor, MI, United States; ^35^Division of Stem Cell Transplantation and Regenerative Medicine, Department of Pediatrics, Stanford School of Medicine, Stanford, CA, United States; ^36^Department of Pediatrics, Golisano Children's Hospital, University of Rochester Medical Center, Rochester, NY, United States; ^37^Nemours/Alfred I duPont Hospital for Children, Wilmington, DE, United States; ^38^UPMC Children's Hospital of Pittsburgh, Pittsburgh, PA, United States; ^39^Department of Pediatrics, University of Texas Southwestern Medical Center Dallas, Dallas, TX, United States; ^40^Department of Pediatrics, Oregon Health & Science University, Portland, OR, United States; ^41^Division of Allergy and Immunology, Department of Pediatrics, Morsani College of Medicine, University of South Florida, St. Petersburg, FL, United States; ^42^Division of Allergy and Immunology, Department of Pediatrics, Johns Hopkins All Children's Hospital, St. Petersburg, FL, United States; ^43^Division of Allergy and Immunology, Department of Pediatrics, Massachusetts General Hospital for Children, Boston, MA, United States; ^44^Department of Pediatrics, University of Wisconsin School of Medicine and Public Health, Madison, WI, United States; ^45^Division of Allergy, Immunology and Transplantation, National Institute of Allergy and Infectious Diseases, National Institutes of Health, Bethesda, MD, United States; ^46^Department of Pediatrics, David Geffen School of Medicine at University of California, Los Angeles, CA, United States; ^47^Allen Institute for Immunology and Department of Pediatrics, University of Washington, Seattle, WA, United States

**Keywords:** primary immune deficiencies, autoimmunity, immune dysregulation, hematopoietic cell transplant, genetics

## Abstract

Primary Immune Regulatory Disorders (PIRD) are an expanding group of diseases caused by gene defects in several different immune pathways, such as regulatory T cell function. Patients with PIRD develop clinical manifestations associated with diminished and exaggerated immune responses. Management of these patients is complicated; oftentimes immunosuppressive therapies are insufficient, and patients may require hematopoietic cell transplant (HCT) for treatment. Analysis of HCT data in PIRD patients have previously focused on a single gene defect. This study surveyed transplanted patients with a phenotypic clinical picture consistent with PIRD treated in 33 Primary Immune Deficiency Treatment Consortium centers and European centers. Our data showed that PIRD patients often had immunodeficient and autoimmune features affecting multiple organ systems. Transplantation resulted in resolution of disease manifestations in more than half of the patients with an overall 5-years survival of 67%. This study, the first to encompass disorders across the PIRD spectrum, highlights the need for further research in PIRD management.

## Introduction

The traditional classification of Primary Immune Deficiency Disorders (PIDD) has consisted largely of patients who present with recurrent, severe, or unusual infections due to defects in immune effector mechanisms. However, a growing proportion of the 344 gene defects now associated with primary disorders of the immune system (1) do not have dominant features of infection; rather, the predominant presentation is with immune-mediated pathology including autoimmunity, autoinflammation, or non-malignant lymphoproliferation. To differentiate this group of disorders from traditional PIDD, we propose that they be called collectively, Primary Immune Regulatory Disorders or “PIRD.” An example prototypic PIRD is IPEX (Immune Dysregulation, Polyendocrinopathy, Enteropathy, and X-linked) syndrome since the principal clinical feature is autoimmune in nature, including autoimmune enteropathy, type I diabetes, autoimmune cytopenias, and immune-mediated dermatitis (2, 3). Patients may also have infections, but these are typically a less prominent feature of the disease. Other disease groups that could reasonably be considered in the PIRD category include Autoimmune Lymphoproliferative Syndrome (ALPS), autoinflammatory disorders such as Familial Mediterranean Fever (FMF), interferonopathies, and Common Variable Immunodeficiency (CVID)-like disease in which patients have hypogammaglobulinemia but autoimmune or inflammatory features dominate their clinical presentation.

Management of PIRD patients is challenging and complex given the frequent need for immunosuppressive therapies that is often in the setting of a co-existing increased infectious disease risk (4). Steroids are often used as an initial therapy but have serious long-term complications. Targeted immune therapies, such as cytokine or small molecule inhibitors, are increasingly available with the advantage of fewer global immune suppressive effects. However, patients with PIRD often do not have an adequate clinical response to immunosuppressive treatment, resulting in referral for allogeneic hematopoietic cell transplantation (HCT) as a potentially curative therapy. To date, there has been limited data on the effectiveness of HCT for the PIRD cohort as a whole. Given the increasing recognition of PIRD cases, we surveyed transplant centers affiliated with the Primary Immune Deficiency Treatment Consortium (PIDTC) in the US and Canada as well as European centers to assemble data from PIRD patients with and without known genetic defects, who have undergone HCT at these sites.

## Methods

A survey was developed by a committee of immunologists, rheumatologists, and blood and marrow transplant specialists who care for patients with PIRD (Supplementary Table 1). The survey was sent via email to all PIDTC sites (*n* = 44, https://www.rarediseasesnetwork.org/cms/pidtc/Learn-More/Participating-Clinical-Centers) (5) and three HCT referral centers in Europe to determine the number and characteristics of PIRD patients treated by HCT at each center. The majority of centers (81%, 33 PIDTC and 3 European sites) responded to the survey. Non-responding centers were contacted at least three times by follow-up emails and/or phone calls. Survey data were collected from January 2017 to October 2017. For the purposes of this survey, a definition of “PIRD” was not provided. Instead, centers were asked to report any patient that had been transplanted specifically to treat clinical features of “immune dysregulation.” Examples and disease categories were provided such as CTLA4, IPEX, rheumatologic disorders, and inflammatory bowel disease. For each patient, the following information was requested: working diagnosis, genetic defect (if known), clinical manifestations, HCT indication, HCT conditioning regimen, donor and hematopoietic cell source, and outcomes following HCT. Clinical manifestations were requested based on categories and the center provided their impression of whether an individual patient had each manifestation. For the analysis, patients were grouped based on gene defects with similar immune mechanisms or by the clinical manifestations. Patients with HLH or genetic defects associated with familial HLH were excluded, as these patients make up a unique group of immune regulatory disorders that are being studied separately. Statistical analysis was performed using Statistical Analysis Software (SAS) v9.4.

## Results

The survey identified 226 patients with PIRD who received HCT between 1982 and 2017 (median year 2011) from 30 PIDTC centers in North America and 3 European HCT centers. Within the cohort, 76% (*n* = 171) had an immune-related gene defect identified in one of 31 genes (Table 1). The remaining patients had clinical features of PIRD, resembling those with known genetic defects, but lacked an identified mutation or had not undergone genetic testing. Patients were grouped into 11 categories based on common clinical features or shared genetic immune pathway defects (Table 1). The majority of patients with an unknown genetic cause were in the CVID group. It is possible that some of these patients would have been found to have genetic defects if current genetic testing approaches had been available at the time of their HCT.

**Table 1:** Disease groups with associated genes or pathways.

**Group**	**Genes/Pathways**	**# of HCT Patients (% of Total)**
APDS	PIK3CD	20 (8.8%)
	PIK3R1	3 (1.3%)
Autoimmunity	C1Q	3 (1.3%)
	Unknown gene	11 (4.9%)
Autoinflammatory	ADA2	2 (0.9%)
	MVK	1 (0.4%)
	PSTPIP1	1 (0.4%)
CID	CD40L	3 (1.3%)
	DOCK8	2 (0.9%)
	MALT1	1 (0.4%)
	RAG1	1 (0.4%)
	ZAP70	3 (1.3%)
	Unknown gene	6 (2.7%)
CVID	TNFRSF13B (TACI)	1 (0.4%)
	Unknown gene	31 (13.7%)
IBD	IL10R	7 (3.1%)
	Unknown gene	1 (0.4%)
Innate	CD18	1 (0.4%)
	IFNGR	1 (0.4%)
	LAD	2 (0.9%)
	STAT1-GOF	3 (1.3%)
	TLR3 and STAT1-LOF	1 (0.4%)
LPD-Like	ITK	1 (0.4%)
	SAP	1 (0.4%)
	XIAP	1 (0.4%)
	Unknown gene	1 (0.4%)
NFKB	IKBKB-LOF	4 (1.8%)
	IKBKG	10 (4.4%)
	NFKBIA	2 (0.9%)
	Other	1 (0.4%)
Tregopathies	CTLA4	13 (5.8%)
	FOXP3	62 (27.4%)
	IL2RA (CD25)	1 (0.4%)
	LRBA	4 (1.8%)
	STAT3-GOF	12 (5.3%)
	Unknown gene	5 (2.2%)
Other^*^	TCF4	1 (0.4%)
	TTC7A	2 (0.9%)

* This group included genes that were associated with syndromic manifestations.

*APDS, activated PI3K delta syndrome; CID, combined immune deficiency; CVID, common variable immunodeficiency; GOF, gain of function; IBD, inflammatory bowel disease, Innate, disorders of innate immunity; LOF, loss of function; LPD, lymphoproliferative disorder, NFKB, nuclear factor kappa-light-chain-enhancer of activated B cells; Tregopathies, T regulatory cell disorders*.

**Figure 1:**
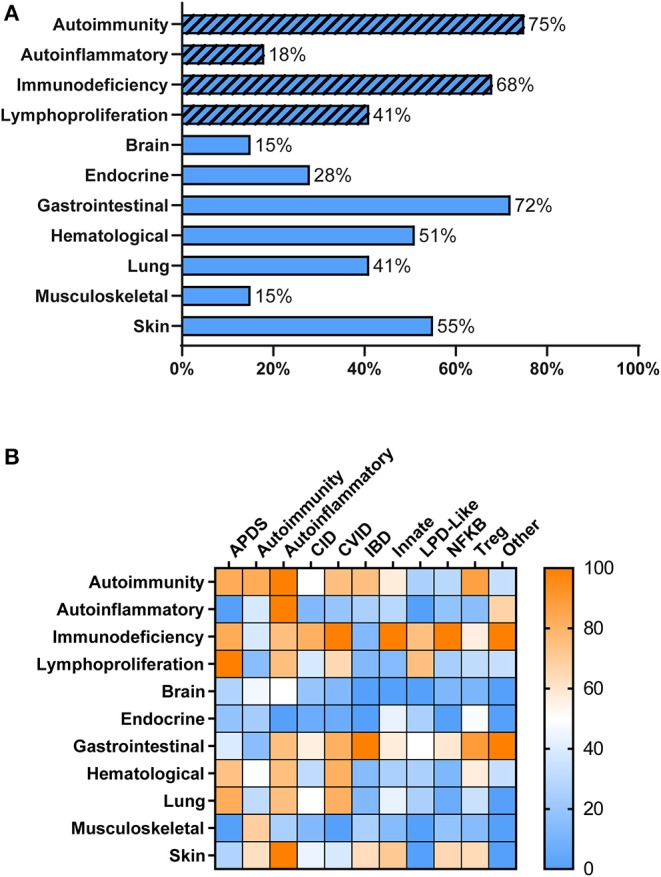
Clinical Manifestations of PIRD Patients. **(A)** PIRD patients have a range of clinical manifestations. Hatched bars represent the type of immune dysregulation [i.e., overactive immune features (autoimmunity, autoinflammation, lymphoproliferation) or underactive immune features (immunodeficiency)] and solid bars represent organ involvement. **(B)** Heat map of the clinical manifestations (rows) in the disease groups (columns). Color indicates the percentage of patients within that disease group that have the particular clinical manifestation. Right bar represents the color scaling. APDS, activated PI3K delta syndrome; CID, combined immunodeficiency; CVID, common variable immunodeficiency; IBD, inflammatory bowel disease; Innate, disorders of innate immunity; LPD, lymphoproliferative disorder; NFKB, nuclear factor kappa-light-chain-enhancer of activated B cells disorders; Tregopathies, T regulatory cell disorders.

Overall, patients with PIRD had a mean age of disease onset of 2 years (median < 1 year, range 0–20 years) with 51% of patients presenting at <1 year of age. As anticipated, individuals with PIRD had clinical manifestations indicative of overactive immunity (i.e., autoimmunity, autoinflammation, lymphoproliferation) co-existing with impaired immune function (i.e., immunodeficiency) (Figure 1A). Virtually all organ systems were affected by immune-mediated pathology, but the gastrointestinal (GI) system was most commonly involved, with 72% of patients reported to have GI symptoms (Figure 1A). Among patients with GI symptoms, enteropathy was the most common GI manifestation (63%) followed by hepatitis (12%). A large proportion of patients had failure to thrive (67%) likely related to GI involvement. Autoimmune cytopenias (51%) were also common in PIRD patients with 16% having hemolytic anemia, 10% having immune-mediated thrombocytopenia, and 21% having Evans syndrome. Skin involvement was also prominent (55%) with the majority of these patients having IPEX or gene defects in the NFkB signaling pathway. Organs less involved in PIRD patients included brain, endocrine organs, and musculoskeletal system.

Within each phenotypic disease group, the clinical manifestations were more variable (Figure 1B). Hematologic manifestations including autoimmune cytopenias, occurred most commonly in subjects having APDS (activated PI3K delta syndrome), CVID, T regulatory cell disorders (Tregopathies), and autoinflammatory gene disorders. Lymphoproliferation was also commonly seen in these groups. Organ specific immune dysregulation also varied by group. Lung disease occurred most frequently in subjects in the APDS, autoinflammatory genes, and CVID groups, while endocrinopathies occurred most frequently in Tregopathies and innate disease categories. Immunodeficiency and infections occurred in >65% of patients in all disease categories except for the autoimmunity and inflammatory bowel disease groups. Autoinflammatory manifestations were not commonly reported in the different disease groups except in known autoinflammatory gene disorders and the “other” category.

All patients (*n* = 226) included in the survey underwent allogeneic HCT to manage PIRD features. The primary indication for transplant was autoimmune manifestations (41%), followed by immunodeficiency (26%), autoinflammation (8%), lymphoproliferative disease (1%), and malignancy (1%). Twenty-two percent of patients had multiple indications for transplant. The median age at HCT was 7 years (range < 1–64 years). Approximately one quarter (24%) underwent HCT prior to 1 year of age and 87% underwent HCT before age 18. The time between the onset of symptoms and transplant ranged from 0 to 58 years with a median of 5 years. The donor source was predominantly bone marrow (65%), followed by peripheral blood stem cells (20%), and umbilical cord blood (14%). Human Leukocyte Antigen (HLA)-matched related donors were utilized in 22% of cases, but the majority received grafts from HLA-matched unrelated donors (53%). Mismatched unrelated donors were used in 18% of cases, haploidentical donors in 4%, and more than 2 donors were needed in 1% of cases (*n* = 3). Conditioning regimens were characterized by the reporting centers as myeloablative (39%, *n* = 87), reduced intensity (36%, *n* = 82), or minimal intensity (8%, *n* = 18). Conditioning intensity was not reported in 17% of cases (*n* = 39).

**Figure 2:**
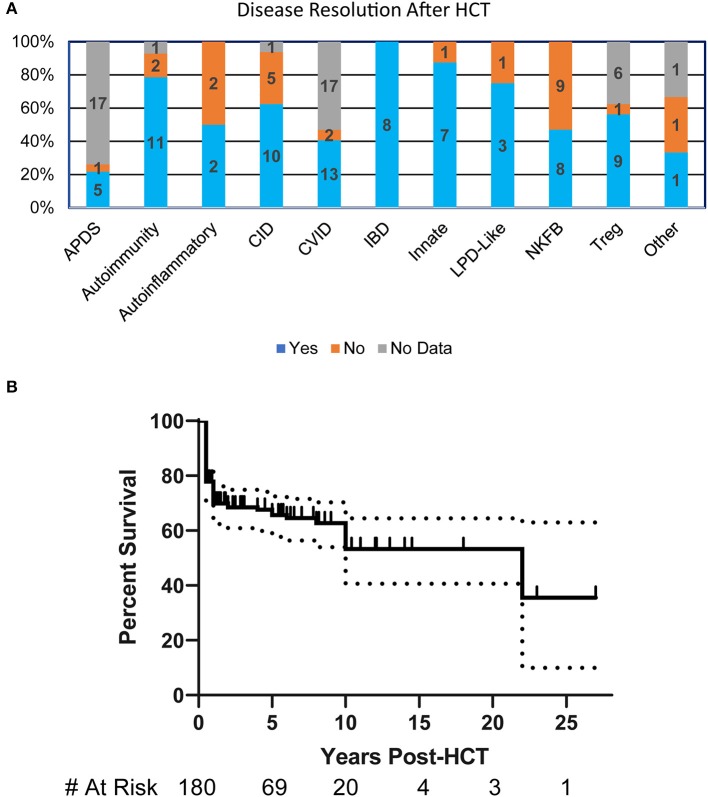
HCT Outcomes in patients with PIRD. **(A)** Bar graph illustrating the resolution of disease at any point post-HCT in each of the disease groups based on percentages within the group. Numbers represent patients in each subgroup. **(B)** Kaplan-Meier estimate of survival in PIRD patients undergoing HCT. Dotted lines represent 95% confidence interval.

More than half (55%) of the patients had resolution of their clinical manifestations after HCT (*n* = 125). Interestingly, all patients in the IBD disease group had complete resolution of their symptoms. Most disease groups had substantial symptom resolution following HCT except for those with NFκB defects where <50% of patients had improvement (Figure 2A). The overall probability of survival at 5-years based on Kaplan-Meier estimate is 67% (95% CI 59–74%) (Figure 2B). Univariate or multivariate analysis suggested, though not reaching statistical significance, that age of disease onset >5 years, being older than 5 years of age at HCT, or undergoing HCT before 2000 were associated with increased mortality. The most commonly reported causes of death were infection (30%), multifactorial causes (18%), and graft vs. host disease (GVHD) (12%).

## Discussion

The is the first study that has collectively reported on HCT for the expanding group of PIRD across a broad sample of treatment centers. The purpose of the study was to summarize recent use of HCT to treat PIRD of all underlying causes in centers specializing in PIDD throughout the US, Canada and selected European centers of excellence. The results suggest that a large proportion of these rare diseases have required HCT and provide preliminary overview of HCT outcomes. While previous reports of HCT survival in selected PIRD genotypes have ranged from 40 to 80%, this is the first attempt to gain a broader overview of HCT outcomes for clinical features of “immune dysregulation” (2, 3, 6–16). For this reason, the study was intentionally broad in scope and captured only a limited data set of key clinical features and outcomes from these patients. The depth of information gathered limited our ability to perform in-depth analyses of specific clinical manifestations and HCT regimens. This is the first study that has collectively reported on HCT for the expanding group of PIRD disorders across a broad sample of treatment centers.

This study highlights the clinical manifestations that prompted a consideration of HCT in patients with a suspected PIRD disorder. Among these, severe GI disease was the most common, but this may reflect the fact that patients with IPEX made up the largest single group of patients reported by centers (62/226, 27%). The study illustrates that HCT can be effective for patients with PIRD; resolution of disease symptoms occurred in at least a portion of the patients across most disease groups, but overall long-term survival remained poor (67% at 5-years) with a large portion of deaths occurring in the first 2 years of life. The survival found in this cohort is similar to that recently published for HCT in a broad spectrum of autoimmune and autoinflammatory diseases from European centers (70% at 5-years) (17). Both studies gathered retrospective data spanning a broad timeframe where there has been significant advances in conditioning regimen, targeted immune modulatory therapies for GVHD, and supportive care. It is possible with current advances in HCT practices that transplantation may be a more optimal therapy to consider earlier in the disease course. Therefore, significant work is needed to identify the types of patients that would benefit most from HCT, to better understand the factors that lead to death after transplant, and to discern potential modifications that could be made to treatment regimens to improve outcomes. Our finding of a trend toward better survival in patients who were diagnosed and treated earlier in the course of disease, suggests that further studies are also needed to learn how to best diagnose and manage this expanding group of complex disorders prior to transplant.

## Data Availability Statement

The datasets generated for this study are available on request to the corresponding author.

## Author Contributions

AC, JL, and TT lead the study design. LB, EA, LN, MJC, MAC, RB, JW, MD, CS, LG, DK, JP, and MP also contributed to the study design. These authors provided patient data AC, JL, EA, SS-S, GU, MS, AG, AS, S-YP, MJ, RM, MJC, CD, JC, SEP, SC, NK, RHB, SP, DC, BO, JB, MAC, SS, BD, LF, CM, EH, DS, KC, KS, JH, NW, MB, GC, FG, IM, HM, MGS, MV, KW, JA, EC, HC, MM, VA, and ES. AC and JL organized and analyzed the data. XL and BL performed statistical analysis. AC, JL, and TT wrote the manuscript. All authors contributed to manuscript revisions, read and approved the submitted version.

### Conflict of Interest

The authors declare that the research was conducted in the absence of any commercial or financial relationships that could be construed as a potential conflict of interest.

## Supplementary Material

Supplementary Table 1 PIRD survey questions.
