# Supplementary material for: Hematopoietic Cell Transplantation in Patients With Primary Immune Regulatory Disorders (PIRD): A Primary Immune Deficiency Treatment Consortium (PIDTC) Survey
Source: Front Immunol. 2020 Feb 21;11:239. doi: 10.3389/fimmu.2020.00239 (PMC7046837; doi:10.3389/fimmu.2020.00239)
Supplement: Supplementary Table 1 — PIRD survey questions. [file Data_Sheet_1.PDF]

Supplementary Table 1: PIRD Survey Questions

Question		Example Pt	1
1	Is there a genetic defect? (yes/no)	yes	
	What is the genetic defect and the functional effect if known (LOF or GOF)?	IPEX, LOF	
	If none, what is the diagnosis (CVID, SLE, Other, etc.)?		
2	Which of the following syptoms did the patient have prior to transplant:		
	Hematologic Cytopenia(s) (yes/no), Please list line affected (plt, hgb, wbc).	no	
	Lymphoproliferation or lympoid hyperplasia (yes/no)	yes	
	Autoimmune/Inflammatory (yes/no)	yes	
	Autoinflammation (yes/no)	yes	
	Lung (yes/no)	no	
	Brain (yes/no)	no	
	Endocrinopathies (yes/no) Please list type (thyroid, diabetes, etc)	no	
	Skin (yes/no)	no	
	GI (yes/no) Please list type (colitis, enteropahty, hepatitis, etc.)	yes, colitis	
	Failure to thrive (yes/no)	yes	
	Immunodeficiency (yes/no)	yes	
	Recurrent/chronic Infection (yes/no)	yes	
	Arthritis, Musculoskeletal (yes/no)	no	
	Other		
3	What was the transplant indication(s)? (ie immunodeficiency, autoimmunity, medical therapy failure, chronic infection, malignancy, bone marrow failure, cytopenias, etc)	immunodeficiency	
4	Please provide the following for the patient		
	year of birth (YYYY, 1900-2017)	2015	
	year of symptom onset (YYYY, 1900-2017)	2015	
	year of transplant (YYYY, 1900-2017)	2016	
5	What type of transplant did the patient receive (BM, UCB, PBSC)	BM	
	Type of donor (MSD, MUD, haplo, MMRD, MMUD)	MUD	
	Matching	10/12	
	Type of conditioning regimen (None, MIC, RIC, myeloablative)	RIC	
6	Did the transplant resolve the indication for transplant? (yes/no)	yes	
	If so, how many months post-transplant	12	
7	Is the patient alive or dead?	alive	
	If alive, how many years or months post-transplant at the present time?	14mo	
	If dead, age of death or year of death?		
	Cause of death?		

List Of Diseases Considered

- IPEX, IPEX-like
- IL2RA
- STAT5B
- PIK3CD, PIK3R1
- CVID with autoimmunity/inflammatory disease
- NFkB signalling defects (NEMO, IKBa, NFKB1, NFKB2, MALT1, CARD11, BCL10)
- C1q
- IL-10, IL-10R
- CTLA-4-haploinsufficiency
- GOF-STAT1
- GOF-STAT3
- LRBA
- XIAP with Inflammatory bowel disease
- ADA2
- TTC7A, TTC37
- IBD (Infantile, Very Early, Early, or other)
- Autoinflammatory (HIGD, CAPS, TRAPS, FMF, etc)
- Interferonopathies
- Rheumatological diseases (JIA, SLE, scleroderma, etc)
- Autoimmune or Inflammatory NOS
- Other immune dysregulation disorders with specific gene defect

Excluding Following Diseases

- Primary HLH
